# Biofilm and wound healing: from bench to bedside

**DOI:** 10.1186/s40001-023-01121-7

**Published:** 2023-04-25

**Authors:** Aakansha Giri Goswami, Somprakas Basu, Tuhina Banerjee, Vijay Kumar Shukla

**Affiliations:** 1 All India Institute of Medical Sciences, Rishikesh, 249203 India; 2 Banaras Hindu University, Varanasi, India

**Keywords:** Biofilm, Bacteria, Wound healing, Quorum sensing, Debridement

## Abstract

The bubbling community of microorganisms, consisting of diverse colonies encased in a self-produced protective matrix and playing an essential role in the persistence of infection and antimicrobial resistance, is often referred to as a biofilm. Although apparently indolent, the biofilm involves not only inanimate surfaces but also living tissue, making it truly ubiquitous. The mechanism of biofilm formation, its growth, and the development of resistance are ever-intriguing subjects and are yet to be completely deciphered. Although an abundance of studies in recent years has focused on the various ways to create potential anti-biofilm and antimicrobial therapeutics, a dearth of a clear standard of clinical practice remains, and therefore, there is essentially a need for translating laboratory research to novel bedside anti-biofilm strategies that can provide a better clinical outcome. Of significance, biofilm is responsible for faulty wound healing and wound chronicity. The experimental studies report the prevalence of biofilm in chronic wounds anywhere between 20 and 100%, which makes it a topic of significant concern in wound healing. The ongoing scientific endeavor to comprehensively understand the mechanism of biofilm interaction with wounds and generate standardized anti-biofilm measures which are reproducible in the clinical setting is the challenge of the hour. In this context of “more needs to be done”, we aim to explore various effective and clinically meaningful methods currently available for biofilm management and how these tools can be translated into safe clinical practice.

## Background

Biofilms have been known since the seventeenth century when they were first described as animalcules by Anton von Leeuwenhoek. But it was not until the early twentieth century that their amazing interaction with wound biology was unraveled. Although bacteria are ubiquitous, their existence as attached colonies enables them to assume multicellular behavior. Heukelekian and Heller in 1940 first observed that a suitable “surface” enables bacteria to grow in colonies, and once an active bacterial slime is established, the biological process is greatly accelerated [1]. Geesey et al. used phase-contrast microscopy and proposed that the slime-enmeshed microbial colonies constituted 99% of functional communities within which most sessile bacteria live [2]. This led to the dismission of the classical paradigm of planktonic bacterial lifestyle which was widely prevalent in the previous century. Electron microscopic studies revealed microbiological variety and the physical arrangement of this shiny, translucent layer [3]. Characklis et al. in 1973 highlighted its tenacity against eradication methods and revealed that bactericidal hypochlorite did not reduce the slime [4]. Although believed to be dreary, dull, flat layers of cells covered with slime, it was the advent of confocal microscopic images, which opened the door to a wider and deeper perspective of wound–biofilm interaction. They have a complex architecture that makes one wonder whether simple prokaryotes could transform into eukaryotic complexities. Bill Costerton [5] coined the term “biofilm” and in 2002, biofilms were first described as a microbially derived sessile community characterized by cells that are irreversibly attached to a substratum or interface or to each other, embedded in a matrix of extracellular polymeric substance (EPS) that they have produced, and exhibit an altered phenotype with respect to growth rate and gene transcription. This led to a significant question “what is the need for bacterial evolution through such intricacies with the ultimate desire for grouped behavior?”

A biofilm confers bacteria certain abilities which are absent in the free-living planktonic form. It provides a microbial home for the colonizing organisms by creating an appropriate physicochemical environment and renders protection from the host, environment, and other competing species. Microbes existing in a biofilm are 1000–1500 times more resistant to antibiotics than in their planktonic state. They also facilitate the uptake of nutrients and the removal of metabolic products through the primitive circulatory system [6]. An important characteristic seen in biofilm bacteria is gene transfer and quorum sensing both of which confer biofilms with distinct properties that provide protection, aid inter-cellular communication and preferentially encourage the growth of beneficial species [7, 8]. Biofilm-related gene expression regulation is yet another mechanism that offers protection against antibiotics. Eventually, the presence of such an “evolved species” makes the wound persistent and chronic. Even with the possession of modern technologies to study these organisms, researchers have realized their incompetence to completely understand the biofilms. The ability of the microorganisms to adapt and attach to any environment poses a great challenge in the treatment of recalcitrant wounds.

## Biofilm: mischief maker in an armored cocoon

The transition of free-floating planktonic forms to biofilm growth involves complex, multiple signals from spatial and temporal reorganization to changes in gene expression. However, this “cocooned” infestation is not necessarily pathogenic. Percival et al., in their review of microbial biofilm, proposed that it is not the dormant, relatively benign, or commensal bacteria that impairs the wound-healing process but the presence of biochemically and genetically upregulated pathogenic biofilm bacteria which do [9]. These colonized resistant benign biofilm bacteria upon upregulation can revert to virulent pathogenic biofilm bacteria causing harm, tissue damage, and finally dissemination. Further interaction of the polymicrobial biofilm community with its surrounding environment creates devastating “hyper-inflammation”, thereby, establishing a chronic and recalcitrant wound-healing process [10]. The direct effect includes the production of destructive enzymes and toxins, whereas the indirect effect promotes a hyper-inflammatory state which slowly brings the wound-healing process more bacteria-centric (controlled by bacteria) rather than host-centric (controlled by the host’s physiological processes). Eventually, it results in an imbalance between the favorable growth factors and the destructive lytic enzymes, free radicals which subsequently affect cell proliferation and wound-healing capability. The persistent inflammation in the wound bed allows for abundant nutrient-rich exudate that helps further the bacterial cause. Furthermore, this hyper-inflammation prevents a Th2 response, a process of development of adaptive immunity, necessary to train the immune system for better recognition and killing.

## The facilitating factors and environment

### A. Host factors

1. **Wound depth**: Although not well understood, studies involving wound samples have shown that ulcer depth is positively associated with an anaerobic environment and proliferation of facultative anaerobic bacteria although the relation is inverse in relation to *Staphylococcus* [11, 12].
2. **Wound duration:** Gardner et al. demonstrated that considering the entire wound microbiome, ulcer duration positively correlated with bacterial diversity and species richness with a relative abundance of Proteobacteria and negatively correlated with a relative abundance of *Staphylococcus* [12].
3. **Local tissue hypoxia:** Microvascular complications lead to tissue ischemia causing a considerable delay in wound healing [13]. Studies have linked miRNAs to angiogenesis and various stages of wound healing. It is believed that tissue hypoxia and low oxygen tension, alter their levels impairing the wound-healing process as evidenced by murine models of ischemic wounds [14, 15]. This hypoxic environment coupled with the presence of necrosed tissue, promotes the proliferation of facultative anaerobes [16].
4. **Immune system:** Chronic wounds exhibit a persistent inflammatory phase, causing physiological changes in the wound bed and predisposing to a wide variety of bacterial species colonization [17]. It is suggested that the downregulation of TLR-2 in injured tissue impedes the immune system and inflammatory response, which causes a reduced chemotactic effect that delays the recruitment of various inflammatory cells [18–20]. The sustained production of pro-inflammatory cytokines, impaired immune cell function, poor angiogenic response, microvascular complications, compromised keratinocytes, downregulation of fibroblast proliferation and migration, and subsequent decrease in production of growth factors associated with wound healing have been reported and implicated as causes of delayed healing in diabetic animal models [21, 22].

### B. Microbial factors

1. **High bacterial diversity**: Dowd et al. introduced the concept of functional equivalent pathogroups (FEP) in which individual members of the biofilm community do not cause disease individually but it is their co-aggregation into an FEP that provides the synergistic effect. This gives the biofilm community the favorable factors necessary to maintain sustained inflammation and infection in the wound [23]. Redel et al. observed that ecological alterations in the wound micro-environment influence the risk of wound infections [24]. Oates et al. concluded that chronic diabetic foot wounds harbored greater eubacterial diversity than healthy skin with *Staphylococcus aureus* being the most common organism [25]. These studies were derived mostly on microbiome-based studies from wound and cutaneous samples of patients with chronic wounds, thus revealing the in vivo situations.

To understand the bacterial diversity, Percival et al. in their review paper reflected on the presence of anaerobes in DFUs and infection [26]. The role of anaerobes in biofilms and their coexistence with aerobic species has been largely under investigation. But current evidence emphasizes the significance of anaerobes in multi‐species biofilm communities [27–29]. The true frequency of anaerobes in surgical wounds remains unclear, largely related to a diverse variety of bacterial culture methods, the types of samples taken for analysis, and the transport media used. However, anaerobes are predominantly seen in DFUs that are deeper, more chronic, and associated with ischemia, gangrene, or a foul odor [30].

Summarizing the available literature, the most encountered bacteria in chronic wound biofilms are the ESKAPE pathogens (*Enterococcus faecium*, *S. aureus*, *Klebsiella pneumoniae*, *Acinetobacter baumannii*, *Pseudomonas aeruginosa*, and *Enterobacter* spp). Others such as coagulase-negative staphylococci and *Proteus* spp. are also involved [31, 32]. While the focus until now had largely been on the diverse bacterial pathogens in chronic wounds, the role of fungi (particularly *Candida* species) in wound biofilms is assuming increased significance [33, 34]. However, it should be emphasized that majority of this literature has been the result of studies on bacteria isolated from the wounds rather than directly studying the biofilm from the wounds.

2) **Microbial load:** It is well documented that wounds are susceptible to infections when the microbial load reaches a critical level or “critical colonization” [35]. However, the concept is contentious. Bendy et al. in 1964 first proposed that microbial numbers play an important role in the non-healing of wounds [36]. This was supported by other studies emphasizing the critical level which was hazardous for the healing process [37, 38]. However, in 1997, Robson et al. in his study documented that healing occurred even in presence of high bacteria count [39]. This observation questioned the concept of the role of microbial load in assessing the risk of wound infection and healing progression. The fact that bacteria are randomly distributed within the wound environment and determining their microbial density is very subjective, revolutionized the idea that microbial number alone should not be used to predict wound infection.
3) **Microbial pathogenicity:** Polymicrobial nature of biofilms in chronic wounds produces a synergistic effect that results in non-virulent bacterial species becoming virulent and causing damage to the host. These synergistic interactions within a biofilm eventually affect the bio-volume and bio-functionality of the biofilm [40]. However, it is the critical control and modulation of gene expression which enables phenotypically different forms of bacteria to survive during adverse external conditions [41]. This concept of biological insurance confers resistance to the host immune response and various antimicrobials [42]. In addition, the synergistic, antagonistic, and mutualistic cooperation between the microbes in the wound bed facilitates a complex yet balanced microbial community that exists in a state of homeostasis with the host wound bed [43, 44]. The stability and long-term survival of this microbial community are possible through the formation of an efficient communication system that can coordinate the function and activities of different species as well as gene expression [24, 45]. To study the notion of biofilm dissemination, Guilhen et al. suggested that biofilm detaches and disperses in response to various environmental and biological signals which helps in their colonization into the surrounding environment [46].

Several virulence traits of *P. aeruginosa* are involved in biofilm formation. Among the surface appendages, type IV pili help in adhesion in biofilm formation, flagella help in maturation of the biofilm, lipopolysaccharide (LPS) layer activates neutrophils to ‘trap’ pathogens thus indirectly protecting itself. Besides, the secretion systems (type III–VI) also help in inflammation and invasion. Among the secreted proteins, EPS of *P. aeruginosa* is also the main constituent of *P. aeruginosa* biofilms. Lipase A and alginate secreted by the bacteria interacts in the extracellular matrix of the biofilm resulting in drug resistance. Several quorum-sensing (QS) pathways regulate the release of further virulence factors such as elastase, rhamnolipids, and exotoxin A for the maturation of the biofilm [47]. Similarly, another biofilm-forming organism *S. aureus* also possesses specific virulence traits like microbial surface components recognizing adhesive matrix molecules (MSCRAMMs), fibronectin-binding proteins, autolysins (AtlA), protein A, biofilm-associated protein (Bap), and teichoic acids that help in adhesion to surfaces and host cells and in maintaining the structural integrity of the biofilm [48].

In this context, an emerging concept of ‘theft biofilm’ should be discussed, where the host skin lipids are ‘stolen’ by the bacteria from the skin wound micro-environment to induce the excessive production of several virulence factors. In particular, researchers have shown that *P. aeruginosa*, possessing the largest biofilm aggregates, is capable of utilizing host lipid factors in the upregulation of the ceramidase system which in turn augments biofilm formation [49].

### C. Environmental factors

Heterogeneity within the biofilm seems mandatory to maintain its ecological stability. Hence, any exogenous and endogenous physiological and biochemical changes will alter the relative microbial competitiveness within the wound and, therefore, alter the homeostasis. The demographic characteristics, personal hygiene of the patient, glycemic control, and previous antibiotic exposure all seem to impact the biofilm and its development [50].

## The clinical significance of biofilms in wounds

### A. Recalcitrant wound healing

Two hypotheses have been postulated to understand the complex pathway that leads to biofilm-mediated recalcitrant wound healing. First, the specific bacterial hypothesis suggests that although heterogeneity and complex microbial diversity are integral to the biofilm in a chronic wound, only a few bacterial species contribute to wound infection and are involved in non-healing wounds. In contrast, the non-specific bacterial hypothesis considers the whole biofilm as a unit and suggests that the complex heterogeneous microflora causes delayed wound healing. These theories are yet to be proven conclusively, as it appears that no one possibility in any given wound at any point in time is responsible for any specific outcome. Their understanding can help us use directed therapies to tackle infection and promote wound healing [9, 26].

There have been studies demonstrating the clinical translation of metagenomics-based data. In a longitudinal, prospective study on the microbiome of diabetic foot ulcer wounds, it was shown that the microbial ‘genetic signature’ of the biofilm clearly regulated clinical outcomes. While variants of *S. aureus* in the wound–biofilm microbiome predicted unfavorable outcomes, commensals such as *Corynebacterium striatum* and *Alcaligenes faecalis* from the wound margins also influenced healing. In addition, wound debridement significantly caused a ‘microbiome shift’ in wound microflora with reduction in low-virulence pathogenic anaerobes for a better outcome, as against the antibiotic treatment [51].

Quantitative estimation of bacterial aggregates from varying depths of the wound surfaces had revealed the localization of *S. aureus* biofilms superficially as compared to those of *P. aeruginosa*, which were found much deeper. The knowledge of this spatial organization of the biofilm microflora further supports the benefit of debridement in its clinical management [52]. Another microbiome shift in wound flora is often observed with the administration of topical and systemic antimicrobials, which causes a relative increase in members of Pseudomonadaceae at the cost of a decrease in Streptococcaceae [12]. The concept of FEPs, clinical interventions causing microbiome shifts in biofilms and actual impact of wound debridement in terms of biofilm management and promotion of wound healing has been better revealed by translation of molecular data to that of clinically relevant outcome.

## B. Antimicrobial tolerance and resistance

Tolerance and resistance to antimicrobial agents is a common property of biofilms. Tolerance to antimicrobial agents refers to the ability of the members of the bacterial biofilm to transitorily withstand lethal concentration of antibiotics or biocides primarily by slowing down the vital processes. Such type of inactive and non-dividing cells is called persister cells. The metabolic processes, which are often the targets of antimicrobial agents, are downregulated in persisters. However, they can revert to their normal metabolic functions and replication rate once the antimicrobial agents are removed. In this relevance, small colony variants (SCVs) of *S. aureus* and *P. aeruginosa* are very good examples for their persistence in host cells. These SCVs have often been isolated in clinical samples thus emphasizing their existence inside the host micro-environment [53].

The resistance of biofilms to antibiotics is impressive when compared with planktonic cells. Evidence suggests that when microbes exist in a biofilm, they can become 10–1000 times more resistant to the effects of antimicrobial agents [53]. Although this fact is well established, its underlying molecular mechanisms are not completely understood. Various mechanisms for developing antimicrobial resistance have been suggested (as detailed below), but it is probably the combination of these mechanisms that provide the outcome.

## Diffusion barrier

The polysaccharide matrix is suggested to act as the barrier which prevents access to the bacterial cell. De Beer et al. concluded that the limited penetration of chlorine into the biofilm matrix is an important factor influencing the reduced efficacy of this biocide as compared with its action against planktonic bacteria [54]. Suci et al. studied the penetration of ciprofloxacin through *P. aeruginosa* biofilms with the help of infra-red spectrometry in a culture-based in vitro model. They found that transport of the antibiotic to the biofilm-substratum interface during the 21-min exposure to 100 microgram/ml was found to be significantly impeded by the biofilm. These results suggest that barriers to drug transport inside bacterial cells may be an important factor in antimicrobial resistance [55]. However, Dunne et al. through an in vitro dialysis chamber-based model simulating infected bioimplants failed to demonstrate sterilization of staphylococcal biofilm even though a combination of vancomycin and rifampicin improved the perfusion of the drugs thereby suggesting an alternate method of antimicrobial resistance [56]. Anderl et al. in their study to investigate the penetration of ampicillin and ciprofloxacin through biofilms in an in vitro model showed that despite full penetration of these antibiotics, there was increased resistance of the wild-type strains to ciprofloxacin and the mutant strains to ampicillin as well as ciprofloxacin, suggesting other mechanisms of antibiotic resistance [57].

## Nutrition limitation

Due to nutrient limitations, mature biofilms show a gradual transition of bacterial growth from slow to no growth [58]. This physiological change accounts for their survival against antibiotics. It was also observed that the sensitivity of both planktonic and biofilm bacteria to antimicrobials increased with increasing growth rate, thereby indicating that a slow growth rate protects the biofilm cells from antimicrobial action [59–61].

## General stress response

Interestingly, recent studies have shown that a slow growth rate of deeper bacteria within the biofilm is not due to nutrient limitation per se, but secondary to a general stress response such as temperature changes, pH changes, and the presence of other chemical agents [62, 63]. This hypothesis is plausible as the stress response is the cause of physiological changes that protect the bacteria from environmental stresses. It has been also shown that RpoS (a gene encoding sigma factor in *Escherichia coli* which regulates the stress response and allows cells not only to survive environmental adversities but also prepares them for subsequent stress) is the central regulator of this response and its deletion results in differences within biofilm cell density [64].

## Quorum sensing (QS)

QS is a cell–cell communication mechanism that synchronizes gene expression in response to population cell density. Davies et al. reported that an inter-cellular signal molecule in the development of *P. aeruginosa* biofilms led to the formation of flat, undifferentiated biofilms unlike the wild-type biofilms, which are sensitive to the biocide sodium dodecyl sulfate [65]. However, it has also been shown that antibiotic resistance remains unaffected in defective QS [66]. Thus, the interpretation of the QS mechanism in the development of antibiotic resistance needs support from further studies.

## Biofilm-specific phenotype

The activation or repression of genes when cells attach to any surface results in the expression of the “biofilm-type increased resistance” phenotype. Induction of multidrug efflux pumps and alteration in outer membrane proteins are some of the acquired protective mechanisms that protect the bacteria from the detrimental effects of antimicrobial agent [67–70].

### Host immune response resistance

The transition from planktonic form to complex biofilm produces small molecules, which increase inflammation and induce host cell death. Although planktonic cells are readily cleared, biofilms reduce the effectiveness of immune cells to overcome the epithelial barrier, host microbiome, and various complement fractions to ensure survival. Overall, biofilms stimulate a unique immune response that is yet to be fully understood.

#### Host immune responses to biofilm constituents

The deep embedment of bacteria helps to evade the host immune system. Instead, the immune system comes first in contact with components of the EPS matrix which is a diverse, hydrated mixture of extracellular DNA (e-DNA, bacterial and host), proteins, polysaccharides, and lipids. Besides being a mechanical barrier, EPS helps elicit the host immune response both in form of immunomodulation and immunogenetic. Some studies have also suggested that bacterial exopolysaccharides block the host immune response by reducing the production of pro-inflammatory cytokines and reactive oxygen species. Besides inactivating innate immunity, they also inhibit complement activation and adaptive immunity [71]. It is important to note that the spectrum of the host response to biofilms and their specific components remains unclear, and more research is needed in this area. As in vitro studies do not take into consideration of the complex wound bed environment, it is also unclear how in vitro results can be translated into clinical scenarios.

#### Host cell response to pathogenic biofilms

Neutrophils play an important role in effectively controlling and eliminating bacterial pathogens. Several mechanisms like phagocytosis, release of antimicrobial peptides, release of reactive oxygen species and formation of web-like chromatin structures called neutrophil extracellular traps (NETs) seem to be involved. These NETs protect against large-sized pathogens including biofilms which are not effectively engulfed by neutrophils alone. NETosis causes release of chromatin and other proteins from the neutrophils in a controlled manner thus clearing the pathogen. Studies conducted on porcine burn wound have clearly demonstrated that biofilms of *S. aureus* ‘skew’ the neutrophils through its leucocidins and diminish the effects of NETosis. Similarly, LPS layer of *P. aeruginosa* also induces the activation of NETs only to protect itself from other invading pathogens and strengthen its biofilm [72].

Pathogenic biofilms weaken the host immune cells through several mechanisms such as immobilizing polymorphonuclear neutrophils (PMNs), decreasing the phagocytic potential of macrophages, inhibiting reactive oxygen species production, and reducing bacterial opsonization. In addition, bacteria have evolved to utilize both PMNs and macrophages to enhance hyper-inflammation, and thus leading to a bacteria-centric immune process in which persistent hyper-inflammation is maintained in the wound bed resulting in inflammatory exudate formation, which keeps on providing nutrition to the microbes [73, 74]. The weakened immune response that fails to translate from innate to a more organized adaptive immunity is ineffective to control and kill the microbes in the long run, contributes to hyper-inflammation, causing collateral damage to host tissue due to heightened levels of matrix metalloproteases, neutrophil elastases, and inflammatory cytokines [75].

## Tools for the detection of biofilms

Biofilms cannot be detected in any wound with the naked eye. The presence of a slimy, shiny, translucent layer on the wound surface is a non-specific finding and at best is a piece of probable indirect evidence of the presence of biofilm. To aid in their recognition, several clinical cues have been identified [75, 76]:

1. Wound failing to heal despite the standard of care, or local infection persisting for more than 45 days
2. Persistence of formation of necrotic tissue and friable granulation tissue in the wound bed
3. Failure of antimicrobial agents to facilitate healing
4. Layer of slime on the wound surface that can be easily removed but rebuilds quickly
5. The wound heals partially only to break down again

These clinical cues should arouse suspicion and help to initiate early biofilm-based wound care, although their actual identification requires advanced laboratory techniques. It must be emphasized here that neither there is any quantifiable marker for biofilm detection nor any objective method to define the areas in a wound affected by biofilm. The molecular methods such as DNA/RNA-based analyses [77] and meta-genomic or more recently whole-genome sequencing [78] are more sensitive and accurate but have their own limitations, the most significant of which is their inability to provide information on whether microbial cells are viable, whether the organisms are in a biofilm or planktonic phenotype. False positive detection of contaminating DNA from the clinical environment (including the patient’s skin, surgical instruments, and gloves) is also a matter of concern.

The discovery of Bap in *S. aureus* as well as other Bap homologs on many other bacterial species is revolutionizing the field of biofilm biomarkers. These proteins are found to be present on bacterial surfaces and are reported to be involved in biofilm formation [79]. Other biofilm matrix components such as cellulose, EPS, and e-DNA can also be used as potential biomarkers and may offer species identification [80–82]. Various other approaches such as proteomics and metabolomics are rapidly expanding due to the ability to study biofilm physiology more closely and accurately [83, 84].

Biofilm imaging technology is an advancing field that provides greater insight into the dynamics and complexities of biofilms. The ability to visualize 3D biofilm images combined with fluorescent staining using confocal scanning laser microscopy (CSLM) helps to visualize biofilms in real-time [85]. Besides these novel technologies, the traditional methods of in vitro culture techniques still exist. The slow-growing persisters may not form colonies under routine culturing conditions and thus cause a false-negative result. Nevertheless, biofilms with a highly heterogeneous population of fastidious strains require specific growth factors for their cultivation.

Even though the field of biofilm diagnosis is fast evolving, routine biofilm characterization and detection have multiple challenges. Therefore, the need for a standardized and reliable method for detection in clinical settings cannot be overlooked.

## Therapeutic options

The biofilm construct, wound micro-environment, and the intrinsic specialties of the biofilm bacteria make the biofilm extremely tolerant to antibiotics and antimicrobial agents. It additionally creates the demand for developing novel anti-biofilm strategies that can be used clinically at the bedside and help improve therapeutic response, and provide a better clinical outcome. Table 1 summarizes various anti-biofilm therapeutic strategies available.

**Table 1:** Anti-biofilm strategies

Anti-biofilm strategies		Mechanism of action	Examples
(A) Inhibition of biofilm initiation			
1	Alteration of physical properties of biomaterials [86–88]	Alteration of hydrophobicity, surface charge	Hyaluronic acid, Hydrogel membranes, fluorinated silica/titanium coatings
2	Alteration of chemical properties of biomaterials [89–91]	Altering the exterior of biomaterial	Ion coatings, biocides, antibiotics
(B) Inhibition of biofilm establishment/Biofilm dispersals			
1	Quorum quenchers [93, 95]	Disruption of the biofilm mode of existence	Meta-bromo-thiolactone, FS3, Daptomycin
2	Anti-Quorum sensing peptides [96]	Attenuate quorum sensing	RNA III-inhibiting peptide
(C) Biofilm eradicators			
1	Destroy extracellular polymeric substances (EPS) [97]	Disruption of protective EPS to expose bacteria to antibacterial agents	Deoxyribonuclease I (DNase I), Dispersin B, Alginate lyase
2	Nanoparticles [98]	Creating artificial channels in EPS	Laser-induced nanobubbles, Magnetic nanoparticles
3	Antimicrobial peptides [99, 100]	Disruption of the cell membrane, inhibition of enzymatic activity	LL-37, Oritavancin
4	Quaternary ammonium compounds (QACs) [101–103]	Disruption of the bacterial membrane–cell lysis	Mono-/bis-/tris-QACs, XF-70, XF-73
5	Antimicrobial lipids [104, 105]	Cell lysis, disruption of electron transport chain, inhibition of bacterial enzymes	Glycerol monolaurate, Docosahexaenoic acid, Eicosapentaenoic acid
6	Bacteriophage [106–108]	Anti-biofilm mediators	PhilBB-PF 7A
7	Natural [109–112]	Variable	1. Plant extracts: Green tea, Dandasa, fresh garlic extract (FGE) 2. Honey: Sidr, Manuka 3. Essential oils: Cumin, Cinnamon oil
8	Novel/modified antibiotics [113–115]	Broad-spectrum antibiotics	Vancomycin-D-octaarginine (V-r8), Pentobra
9	Others [116, 117]	Adjuncts to enhance the sensitivity of conventional antibiotics	1. Electrochemical treatment: damage bacterial membrane 2. Cryogenic freezing

### Therapies targeting bacteria

These modalities target microbial structure and function. The mechanisms can range from direct toxicity to the bacterial cell to inhibition of their enzymes and bacterial signaling pathways.

#### Bacteriophage therapy

Bacteriophages are viruses and the natural predators of bacteria. Their ability to negate the protective biofilm stems from these proposed mechanisms:

- High host specificity thereby preserving beneficial bacterial flora
- Production of phage-encoded enzymes (polysaccharide depolymerase, and alginase) disrupting the biofilm matrix
- Its intrinsic ability to multiply within the bacterial host cell and liberate new virus particles by bacterial cell lysis.
- The cell kill is highly specific and the phage population also goes down as soon as the bacterial population decreases.

Studies on animal wound infection models have demonstrated positive results in the early phases of biofilm formation although the beneficial effect was not sustained in well-formed biofilm [118]. The effect of bacteriophage in a mouse wound model against multidrug-resistant *P. aeruginosa* showed promising outcomes [106]. The phage therapy was then tested on patients with non-healing infected wounds that showed significant improvement in wound healing [119]. Needless to say, the use of bacteriophages as therapeutic agents is yet to be widely accepted. The added benefit of their synergism with concurrent antibiotic use cannot only enhance bacterial killing but is also likely to reduce antibiotic resistance.

#### Nano-antimicrobials and metals

The ability of nano-formulations to cross the biofilm barrier and overcome antimicrobial resistance has increased their popularity in recent years. Besides having an intrinsic antimicrobial activity (such as silver), these also target biofilm matrix and enhance the effect of other modalities (magnetic hyperthermia-based technology). Both in vitro and in vivo studies have demonstrated that silver inhibits both early and mature biofilms [120, 121]. The broad-spectrum antimicrobial ability of these formulations comes from their ability to bind to bacterial structures and destabilize the intermolecular adhesion bonds. Besides using nanoparticles, recent work utilizes the use of nanohybrid enzymes with the aim to activate reactive oxygen species [121, 122]. However their cytotoxicity at high concentrations precludes their clinical use at present. Nonetheless, their physical ability to penetrate the dense matrix with a low likelihood to develop resistance makes them effective against biofilms.

Besides silver, other metals such as cerium and gallium also demonstrate anti-biofilm effects. They interfere with the formation and maturation of biofilm. Consequently, these can be used as a topical application in wound care, thus disrupting and preventing biofilm formation [123]. However, with a handful of approved products, further research is needed for the clinical use of these metals as effective armaments against biofilms.

Another method for increased delivery of antimicrobials to the wound site is through the implantation of biomaterial containing the desired antimicrobial. This method of local and sustained delivery of antimicrobials has been successfully used in preventing biofilm-related wound complications, especially infections in bones [124]. The presence of the antimicrobials at the site of the wound affects the wound micro-environment leading to disruption of the biofilm and initiation of the proliferative phase and healing.

#### Blue light therapy

Several studies have demonstrated the positive benefits of photo biomodulation in wound healing. Although biological mechanisms are yet to be understood completely, studies have shown that visible light between the 400 nm and 500 nm wavelengths has an antimicrobial and anti-biofilm effect [125]. Halstead et al. in their in vitro study tested blue light against planktonic and biofilm bacteria and showed significant bacterial sensitivity to the blue light treatment [126]. It is interesting to note that Gram-positive bacteria are less susceptible and the effect on the older biofilm is still a matter of debate. The ease of administration, minimal side effects, action against a wide variety of microorganisms, and low potential for tolerance, makes them propitious in the management of chronic biofilms.

#### QS inhibitors

QS is an important signaling system consisting of oligopeptides which are released in the extracellular fluid and facilitate cell-to-cell communication in bacterial colonies. QS is responsible for maintaining bacterial population density and virulence factor production [127]. Inhibiting these pathways can prevent biofilm formation and reduce bacterial virulence. Studies have shown that chlorogenic acid decreases bacterial load and accelerates healing in a mouse wound model of *P. aeruginosa* infection via QS [128]. In *S. aureus*, QS has been shown to be inhibited by RNAIII inhibiting peptide (RIP) and its derivatives [129, 130]. QS inhibitors due to their marked synergistic effect with antibiotics can be used as adjuncts to increase the susceptibility of biofilms to antimicrobials [134]. However, their toxic effects on the host cells at working concentration [127, 132] and their reduced efficacy in the in vivo model limit their clinical use at present [133]. In this context, a polyphenolic phytochemical, curcumin, has been extensively studied as an anti-biofilm agent. Curcumin acts by inhibiting the QS systems and disrupts biofilm formation by inhibiting bacterial adhesion to host receptors [134]. Several nano-formulations incorporating curcumin are available for applications both on wound surfaces and on implantable devices to prevent biofilm formation.

#### Matrix-degrading enzymes

Biofilm matrix degradation is yet another promising anti-biofilm strategy. The use of DNAase I, Dispersin B (DspB), and a-amylase to degrade complex biofilm structure allows for increased antibiotic penetration and therefore increases its efficacy [135, 136].

This novel biofilm degrading strategy not only inhibits biofilm formation but also disrupts the mature biofilms of *S. aureu*s, *Vibrio cholerae*, and *P. aeruginosa* [137]. However, the cost of synthesizing pure enzymes for clinical application makes it expensive and limits their clinical use. Nonetheless, combining biofilm matrix-degrading enzymes and antibiotics is a highly effective tool for removing biofilms from recalcitrant wounds [138].

#### Antimicrobial peptides and natural compounds

Antimicrobial peptides are positively charged, amphipathic peptides, 15–30 amino acids in length that can be produced by bacteria and fungi. They bind to negatively charged structural molecules on the microbial membrane and thereby exert a broad spectrum of antimicrobial activity [139]. The major advantage is their ability to act on slow-growing, non-multiplying bacteria as encountered in biofilms. The ability to modify their primary amino acid sequences to enhance their effectiveness and stability makes them attractive anti-biofilm agents [140]. However, their increased susceptibility to body fluid pH, proteolytic activity, and ionic strength makes their clinical application challenging.

Natural and plant-based derivatives have also been used as preventive measures against biofilms. In this regard, the antibacterial effects of honey, both sidr and manuka need mention. The antibacterial effects are presumed to be multifactorial and are thought to be due to the substantial content of dicarbonyl methylglyoxal (MGO), bee defensin-1, a number of phenolic compounds, and complex carbohydrates. Antimicrobial effects exerted by the osmotic effect of high sugar concentration, low pH, and the presence of hydrogen peroxide produced by bee-derived glucose oxidase, are other mechanisms of its antibacterial activity [123]. Furthermore, manuka honey has been shown to affect gene expression in multi-drug-resistant *Staphylococcus aureus* (MRSA) [108].

#### Ultrasonic treatment

Although low-frequency ultrasound is not effective alone in killing biofilm-growing bacteria, it can be combined with antibiotics to enhance antibiotic transport across the biofilms by enhancing the sensitivity of biofilms to antimicrobial agents [141]. Studies have shown that this combination helps in the increased killing of *P. aeruginosa* and *S. aureus* associated biofilms and those caused by drug-resistant *E. coli* [142, 143]. Employing ultrasonic therapy in the management of non-healing wounds is a promising non-invasive means to decrease bacterial bioburden.

#### Electrical and electrochemical approaches

Recent years have ignited the interest in electroceuticals and the effect of electrical current in various stages of wound healing [139]. Human studies have shown that electrical stimulation increases cutaneous perfusion and accelerates wound healing [144]. In a study by Banerjee et al., the growth of P. aeruginosa was markedly arrested in the presence of wireless electroceutical dressing (WED), which in the presence of wound exudate gets activated to generate an electric field. Due to its ability to produce ROS, biofilm thickness was decreased and the activity of quorum-sensing genes was repressed [145]. Similarly, another study demonstrated the ability of WED to disrupt biofilm aggregates and accelerate wound closure by restoring skin barrier functions [146]. These electroceuticals provide novel therapeutic options to improve wound outcomes by enhancing re-epithelization and disrupting biofilms. Its low cost, better safety profile, and long shelf life provide added advantages to its use.

### Therapies targeting wound micro-environment

#### Modification of local pH

The wound bed pH shifts from acidic to alkaline to neutral and then again acidic as the wound heals [147, 148]. Studies have shown that failure of most acute and chronic wounds to heal is correlated with alkaline pH of 7.15 to 8.9 [138, 149]. The arduous wounds which are stalled due to a prolonged inflammatory phase are also subjected to increased protease activity that is pH dependent. Acidification of wounds using topical acetic acid [150], polyacrylic acid, and polycarboxylate vinyl resins have been employed to study wound healing. It is argued that wound acidification being an adjuvant to healing, controls *P. aeruginosa*, which is present in 40% of chronic wounds and is often resistant to antimicrobial therapy [151].

#### Negative pressure wound therapy (NPWT)

Negative pressure wound therapy (NPWT) applies continuous or intermittent sub-atmospheric pressure to the wound surface. Currently, it is a standard of care in difficult wound management. NPWT may assist wound healing by increasing tissue perfusion and help in the production of granulation tissue besides reducing exudates, edema, and bacterial contamination [152]. Recent work suggests that NPWT with the instillation of antimicrobials such as diluted hypochlorous acid contributes to a significant reduction in wound bioburden and thereby shows promising results in wounds with mature biofilms [153]. With the added advantage of absent bacterial resistance development, this technique in combination with topical antiseptics is ideal in the management of difficult-to-heal wounds.

#### Hyperbaric oxygen therapy (HBOT)

It is well known that persistent hypoxia in chronic wounds limits healing. HBOT is an evolving therapy in which 100% oxygen above atmospheric pressure is supplied to the tissues for a defined period with the aim to increase the partial pressure of oxygen in the circulation and thereby increase its delivery to the wound bed. It aids wound healing by improving oxygenation, decreasing inflammation, and enhancing neovascularization [154]. Its positive effect on reducing bacterial biofilms both in vitro and in vivo has also been demonstrated [155]. This may be due to its antimicrobial effect via inducing oxidative stress and host immune system modulation apart from acting synergistically with the antibiotics and thereby enhancing its effects [156, 157]. With almost no likelihood of the development of bacterial resistance, the efficacy of HBOT as an adjunct therapy against biofilms is promising.

#### Surfactants

Surfactants have the capacity to unite compounds with different polarities and reduce the surface tension of the surrounding medium and thereby decreasing their ability to stick together. Besides being used as wound scrubs surfactants can also be used as carriers of antimicrobials. In comparison to the standard silver sulfadiazine cream, surfactants have pro-healing effects on full-thickness skin wounds [159]. Surfactant polymer dressing has been shown to decrease the growth rate of both Gram-positive and Gram-negative organisms, but the resultant effect was mostly bacteriostatic [160]. Surfactants work by disrupting the EPS matrix and converting the biofilm bacteria to planktonic phenotype. This makes bacterial removal easier from wound surfaces and their susceptibility toward antibiotics when used in combination. These molecules can be used to coat dressings and sutures and have fewer chances of developing resistance.

### Therapies targeting bacteria and chronic wound micro-environment

#### Probiotics

The use of live bacteria for achieving health benefits ranges from simple prevention of viral gastroenteritis to the treatment of inflammatory bowel disease. With their immunomodulatory role and ability to replace biofilm-growing pathogens, their use is being considered for the prevention of biofilm formation. Walencka et al. in their study to evaluate the ability of the *Lactobacillus acidophilus*-derived substances to inhibit *S. aureus* and *S. epidermidis* biofilms concluded that inhibition of bacterial attachment and biofilm disruption occurs by influencing cell-to-cell and cell-to-surface interactions [160].

Furthermore, Sadowska et al. observed the antagonistic effect of bacteriocin-like inhibitory substances produced by *L. acidophilus* against the *S. aureus* strains [161]. Varma et al. investigated the anti-infective properties of Lactobacillus fermentum by co-incubating with *S. aureus* and* P. aeruginosa* and observed growth inhibition, increased cytotoxicity, and decreased biofilm formation [162]. Although the results of laboratory studies are promising, there is still a long way to identify the ideal probiotic that can be used clinically as an anti-biofilm tool.

#### Mesenchymal stem cells

Mesenchymal stem cells (MSCs) due to their antimicrobial effect hold tremendous potential for wound infection management. These exert anti-infective effects through both direct and indirect mechanisms. Their ability to secrete antimicrobial peptides as well as modulate pro- and anti-inflammatory immune responses have aroused interest in their therapeutic potential in biofilm-laden wounds [163]. Probably, the most compelling evidence is derived from the article by Johnson et al. who studied the effects of MSC administration in canine models of biofilm-infected wounds, and concluded that the best outcome was found in the co-administration of activated MSC with antibiotics. Furthermore, repeated systemic administration of activated MSC had better bacterial clearing and wound healing [164]. Wood et al. demonstrated that human adipose tissue-derived mesenchymal stem cells (AT-MSCs) inhibited the growth of *S. aureus* and* P. aeruginosa*, which was attributed to secretion of antibacterial factors, enhanced phagocytosis and reduced bacterial adhesion [165]. Their attractive differentiating potential and ability to speed up the wound-healing process by promoting angiogenesis and reducing scar formation makes them a promising tool. However, heterogeneity in their preparation, suboptimal wound bed preparation, cell viability, and the need for larger controlled clinical trials for ensuring safety, preclude their widespread clinical use.

## The current consensus for the management of wound biofilms

Since the biofilm is invisible to the naked eye, identification of biofilm “clinical cues” guides an astute clinician to initiate biofilm-based multifaceted treatment early. The presence of a shiny, slimy layer on a non-healing wound bed that reforms rapidly after its removal and does not respond to standard wound care treatment and antimicrobial intervention is arguably the best indirect evidence of the presence of biofilm in the wound. However, the World Union of Wound Healing Societies (WUWHS) position statement indicates that ‘all non-healing chronic wounds potentially harbor biofilms’ and insists that treatment of such wounds should be directed towards disruption of biofilms and prevention of their reformation [166]. Another consensus document also indicates similar observations and suggests a holistic approach [167]. However, it should be acknowledged that the majority of the studies on the management of biofilm-related wound infections have shown a reduction in biomass or bioburden in wounds rather than eradication of the biofilms. That is to say, the complete eradication of biofilms is extremely difficult. Therefore, a pertinent question is whether better results in wound healing are expected after reduction or after the complete eradication of biofilms. While studies demonstrate that responses to treatment greatly increase even after the reduction of the size of biofilms, recurrence or reformation of biofilms poses a real challenge. As biofilm formation involves a constant balance between the planktonic bacteria and the biofilm-associated bacteria, it would be wise to speculate that the reduction of either population of cells helps to tilt the balance towards the host’s immune factors. However, as bacterial evolution has been faster than expected, there might be other emerging strategies to outsmart this approach. Until further evidence comes in support, reliance on a holistic approach is a better mode of tackling wound biofilms.

**Fig. 1:**
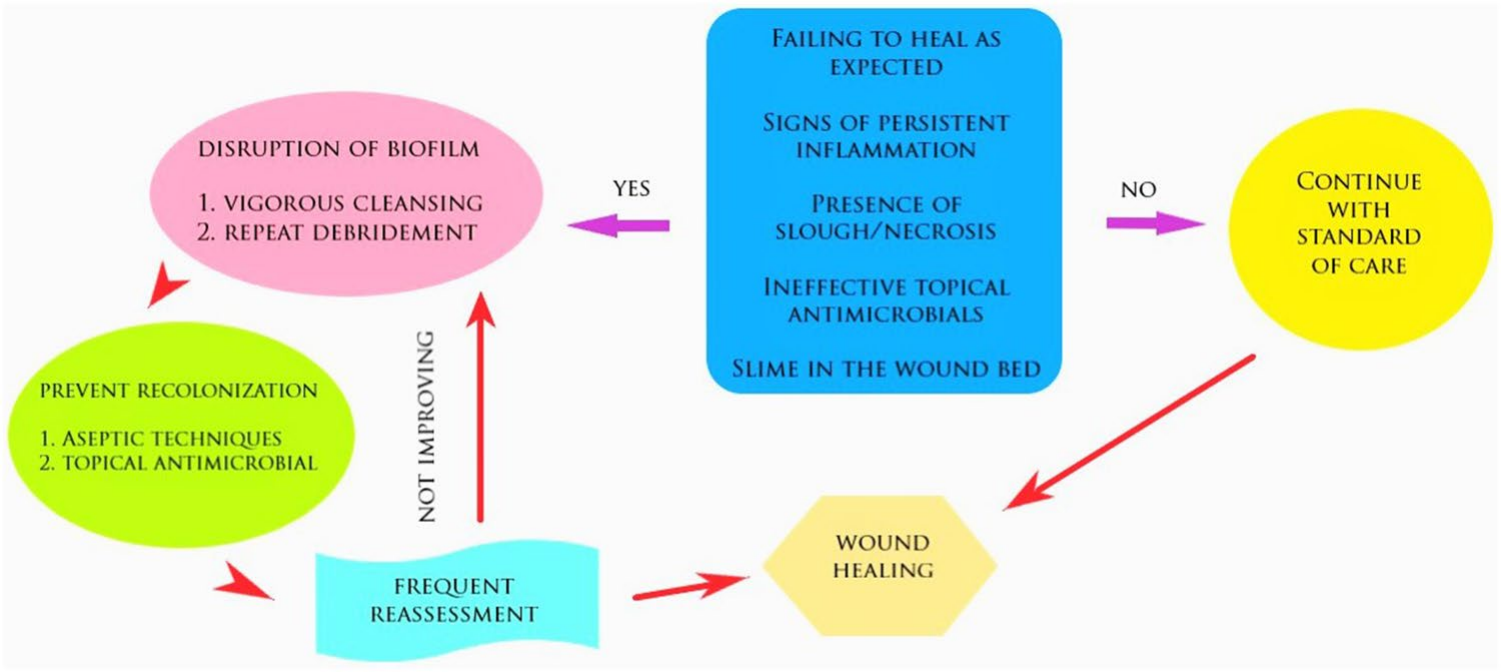
Biofilm-based wound care (BBWC): schema of workflow

The biofilm-based wound care (BBWC) is the holistic approach to biofilm management with an emphasis on initial aggressive debridement and cleansing to reduce the biofilm burden as well as increase antimicrobial susceptibility [168]. The aim is to step-down or bulk up the treatment depending on the healing progression. Once the necrotic, devitalized tissue is removed and the wound bed is prepared, the step-down process ensures the prevention of microbial recontamination and subsequent biofilm reformation. This can be achieved using topical antimicrobials and barrier dressing. In case the wound seems still recalcitrant after 4 weeks of the chosen treatment, the patient and the wound should be reassessed and an alternative treatment strategy should be planned (Fig. 1).

### A. Prevention of biofilm formation

Once the early presence of biofilm is suspected, a proactive approach should be considered to reduce its burden and maturation. The newer anti-biofilm agents can specifically target the early stages of biofilm formation.

#### Prevention of attachment

Anti-adhesion agents such as mannosides, pillicides, and curlicides have shown very promising results in in vitro studies [169–172]. Other agents such as lactoferrin, ethylene diamine tetraacetic acid (EDTA) [173], xylitol, and honey have been shown to cause bacterial destabilization and block the attachment [174]. Other agents which disrupt biofilm EPS (such as EDTA) [168, 170] and interfere with signal transduction mechanisms (such as farnesol, Iberin, aioene, and manuka honey) [175] also prevent biofilm formation and stabilization.

#### Prevention of colony formation and biofilm maturation

Bacteriophages, nanoparticles, antimicrobial peptides, anti-biofilm polysaccharides, and EPS degrading enzymes, all exert a significant inhibitory effect on micro-colony aggregation and biofilm maturation.

#### Biofilm dispersion

This strategy is based on the principle that dispersion forces the biofilm to assume planktonic phenotype making them more susceptible to the combined administration of antimicrobial agents. Ma et al. exploited the use of biofilm dispersal protein, thereby providing a new tool that can facilitate biofilm dispersion [171, 172]. Studies have also shown that d-amino acids promote biofilm disassembly by disrupting adhesive fiber interactions [178]. Another biofilm-disassembly molecule, Norspermidine works complementary to D-amino acids [179], thereby making these useful in anti-biofilm therapy.

### B. Disruption of existing biofilm

For the wound-healing process to progress smoothly, the wound bed must be well perfused, moist, free of necrotic, dead tissue, and clear of infection. Meticulous wound care with regular cleansing, debridement, and barrier dressing can help extirpate the obstinate biofilm and promote healthy granulation tissue formation and re-epithelization.

#### Regular cleansing

Regular wound irrigation should be part of routine wound management. This is to remove the necrotic material and reduce bacterial load. Low-pressure irrigation using a bulb syringe is sufficient for most wounds. For highly contaminated wounds, high-pressure pulse irrigation using sterile saline should be considered.

#### Repeated debridement

Wound debridement facilitates the separation of necrotic tissue from the wound bed. This can be accomplished by various means such as sharp, mechanical, autolytic, and enzymatic debridement [180]. Sharp debridement, although effective and rapid to reduce the bacterial load and stimulate healthy granulation tissue, has the major disadvantage of being painful. Autolytic debridement is the natural method in which proteolytic enzymes in wound fluid remove the necrotic tissue from the wound bed. This natural process can be augmented with the use of semi-occlusive dressings which keeps the wound moist for a long time. Although easy and feasible, the major drawback of this technique is the time taken to produce satisfactory results and the high risk of anaerobic growth which requires frequent monitoring.

Enzymatic debridement is yet another way to digest the proteins in dead nonviable tissue while preserving the healthy tissue underneath. Commercially available collagenase and papain are two widely used agents. Although they are slow and effective in wounds with minimal necrotic tissues, these are usually used in adjunct to surgical debridement [181].

### C. Prevention of biofilm reformation

Once the wound bed is adequately prepared, antimicrobial/anti-biofilm agents should be applied locally to inhibit biofilm reformation. Several antimicrobials such as acetic acid, honey, iodine, polyhexamethylene biguanide (PHMB), and silver have been used in this regard. Although used synonymously, antimicrobials are broad-spectrum agents that are bactericidal or bacteriostatic to the microbes whereas anti-biofilm agents are novel compounds that act against biofilm at various stages of its formation [182].

As discussed above, one of the most important characteristics of biofilms is their increased tolerance to antimicrobial agents. Treatment based on laboratory-derived antimicrobial susceptibility tests may not always correlate with therapeutic success. Topical application provides high local concentrations by delivering antibiotics directly to the site of infection with low or even undetectable serum concentrations, thus avoiding systemic side effects. Topical antibiotics are also beneficial in an avascular area where parenterally administered antibiotics cannot easily reach. Furthermore, topical application may decrease the chances of developing antimicrobial resistance [183]. In this context, antibiotic therapy may have a role in the treatment of established biofilm-associated infections and even as prophylaxis to prevent infection in certain circumstances [183].

Much of the evidence for topical antimicrobial is derived from in vitro studies and due to the large disparity between testing conditions and intended application, most of these anti-biofilm strategies fail when used in vivo [184]. While delivering antimicrobials topically, the concept of minimum biofilm eradication concentration (MBEC) should be kept in mind. Although MBEC is believed to be lower when the antimicrobial exposure time is longer [185], further studies are needed to confirm whether MBEC for in vitro studies translates similarly in clinical infection.

### D. Reassessment

It is an important aspect to determine the success of biofilm treatment. All the initial cues which led to the suspicion of biofilm should be reviewed. The parameters such as reduction in local signs of infection and the decrease in slough are important determinants of successful wound healing. In addition, it is suggested that all the treatment modalities should be given for at least 2 weeks before deciding on their efficacy [71].

## The road ahead

Although extensive data arising out of in vitro and in vivo animal research do demonstrate various mechanisms by which this slimy layer interferes with the wound-healing process [186–189], most of the research studies discussed above are in the experimental phases barring a few which have been successfully introduced in the patient care. The real question remains how to detect biofilms in wound beds in real life and how much wound beds should be involved by biofilms to cause a significant delay in clinical healing. To date, there are little data to suggest to what extent biofilm needs to be present to negatively impact healing. A non-invasive technique has been described that creates a “biofilm map” in the wound bed by “blotting” the wound and mapping it using a specific dye solution to tag the free DNA shed by the biofilm in the wound [190]. It has given considerable clues to localizing biofilm on the wound surface and predicting wound behavior in terms of slough development in subsequent weeks depending on the extent of surface area stained by the dye. But more research is indicated for its commercial use. Since no gold standard technique exists for visualization and measurements of biofilm in wounds, bench research forces us to reconsider whether the laboratory observations can be translated into clinical practice. Evidence-based practice can only be guided by clinical research, which at present is inadequate in substantiating the in vitro anti-biofilm mechanisms tested in the laboratories. Therefore, well-designed clinical trials are the need of the hour to test laboratory evidence and translate these novel techniques to reach the patient’s bedside.

## Conclusion

The laboratory and clinical evidence now establish that the bacterial biofilm is a major potentiator of wound intractability and delayed healing. The pathogenesis is thought to be multifactorial and involves a diverse species of microbes and their intricate interaction with host cells in the wound bed micro-environment. More needs to be understood to detect and reverse the effects of biofilms in wounds. The in vitro experiments are mostly research-based and may not have significant anti-biofilm effects in real-life scenarios. Moreover, most novel diagnostic tools are not clinically available. From a therapeutic perspective, the multimodality approach is currently being strengthened with the search for various anti-biofilm options. Although these recent laboratory developments are promising, translational research is the need of the hour to have a potential impact on wound health, long-term morbidity, and quality of life. Even with the mounting scientific evidence, clinical diagnosis is still limited by the so-called “diagnostic” clinical cues and mechanical debridement along with topical antimicrobial therapy remains the pillars of wound–biofilm management.

## Data Availability

Not applicable.
